# Structural changes in hand related cortical areas after median nerve injury and repair

**DOI:** 10.1038/s41598-018-22792-x

**Published:** 2018-03-14

**Authors:** Per F. Nordmark, Christina Ljungberg, Roland S. Johansson

**Affiliations:** 1 Department of Integrative Medical Biology, Physiology Section and Umeå center for Functional Brain Imaging, Umeå University, SE-901 87 Umeå, Sweden; 2 Department of Surgical and Perioperative Sciences, Hand and Plastic Surgery, Umeå University, SE-901 85 Umeå, Sweden

## Abstract

Transection of the median nerve typically causes lifelong restriction of fine sensory and motor skills of the affected hand despite the best available surgical treatment. Inspired by recent findings on activity-dependent structural plasticity of the adult brain, we used voxel-based morphometry to analyze the brains of 16 right-handed adults who more than two years earlier had suffered injury to the left or right median nerve followed by microsurgical repair. Healthy individuals served as matched controls. Irrespective of side of injury, we observed gray matter reductions in left ventral and right dorsal premotor cortex, and white matter reductions in commissural pathways interconnecting those motor areas. Only left-side injured participants showed gray matter reduction in the hand area of the contralesional primary motor cortex. We interpret these effects as structural manifestations of reduced neural processing linked to restrictions in the diversity of the natural manual dexterity repertoire. Furthermore, irrespective of side of injury, we observed gray matter increases bilaterally in a motion-processing visual area. We interpret this finding as a consequence of increased neural processing linked to greater dependence on vision for control of manual dexterity after median nerve injury because of a compromised somatosensory innervation of the affected hand.

## Introduction

Despite the best available surgical repair, an injury transecting the median nerve typically causes lifelong disability characterized by loss of fine sensory and motor hand functions because of an inadequate reinnervation of the hand^1–4^. Several factors contribute to the defective reinnervation including loss of primary sensory and motor neurons, misdirected reinnervation with respect to location and type of end-organs, and abnormal conduction and stimulus coding properties of the reinnervated sensory neurons^5–10^. Primarily due to an imperfect tactile reinnervation of the fingertips, the affected hand cannot perform certain everyday tasks, especially those require fine finger dexterity. This implies restrictions in the diversity of the natural manual action repertoire, restrictions that also affect bimanual activities.

Inspired by recent findings on activity-dependent structural plasticity in the adult brain^11,12^, we asked whether the imperfect reinnervation and the resulting deterioration in fine dexterity after repaired median nerve injuries can drive structural changes in the brain. Regional gray and white matter changes of varying complexity have previously been associated with adaptations to peripheral conditions affecting perceptual and movement abilities as well as skill learning^13–20^. Structural changes expressed as increases or decreases in gray matter volume are considered to be a consequence of changes in axon sprouting, dendritic branching, synaptic density, glial volume and regional vasculature, while changes in the white matter are considered to reflect activity-dependent adaptations of the myelination of axonal fiber tracts^11,12^.

We used voxel-based morphometry (VBM)^21^ to investigate structural changes in the brains of 16 right-handed nerve-injured adults. Sixteen healthy individuals matched by age, gender and handedness served as control group. The nerve-injured participants had more than two years earlier suffered sharp complete transection of the median nerve in their left (n = 11) or right (n = 5) distal forearm followed by microsurgical repair within 24 hours. Importantly, at two years after such an injury, most of the attainable recovery of hand function has occurred^3,22,23^.

We expected that activity-dependent structural changes might occur in the hand area of the primary sensorimotor cortex contralateral to the injured side since experimental peripheral nerve lesions in animals can cause substantial functional reorganizations both in contralesional primary somatosensory area (S1)^24–27^ and primary motor area (M1)^28–31^. For each group of nerve injured participants (left and right injured) we searched for structural effects on gray matter volume in contralesional S1 and M1 by applying a small-volume correction (SVC) analysis that encompassed both these cortical areas. That human imaging studies indicate that tactile stimulation of fingers can activate the contralesional S1 after peripheral nerve injury and reinnervation^17,32,33^, does not necessarily imply that S1 is structurally unaffected.

To seek for structural effects beyond S1 and M1 we applied whole brain analyses addressing both gray and white matter. We expected that brain areas functionally associated with higher-level planning and control of hand actions might be affected because of altered neural processing linked to changes in hand use after median nerve injury. In these analyses, we looked for effects that would depend on side of injury as well as effects that would be independent of side of injury. To that end, by contrasting the left- and the right-side injured groups we addressed effects that would depend on side of injury and by contrasting all injured participants and the controls we looked for effects that would be independent of side of injury. We expected that potential effects independent of the side of the injury could be both bilateral and lateralized in a hemispherical asymmetrical manner given that hemispherical specialization exists in planning and control of manual praxis^34–37^.

## Results

We first present characteristics of the participants with median nerve injury, and then report structural effects on gray and white matter in the brain.

### Characteristics of the nerve injured study participants

The median age of the injured participants at the time of the injury was 34 years (range: 9–60). At the time of the study the median age was 44 years (range: 18–64) and for the control participants it was 44 years (19–62). On average, the study took place 8 years (2–17) after the injury episode. There were no significant differences between the eleven left and the five right-side injured participants regarding age at the time of injury (median: 34 and 35 years, respectively), age at the time of the study (44 and 45 years), and the time from injury to the time of the study (6 and 9 years; P > 0.65 in all instances, Mann-Whitney U test). Injuries to tendons occurred in all 16 participants and injuries to arteries in a few (Table 1). There was no statistically significant difference between the eleven left-side injured and the five right-side injured in the number of such injuries (P = 0.11; Mann-Whitney U test).

**Table 1:** Injured structures and questionnaire scores.

	1	2	3	4	5	6	7	8	9	10	11	12	13	14	15	16
BR																■
FCR							■		■		■		■			■
PL	■				■	■	■		■	■	■	■	■		■	x
FCU										■					■	■
FPL												■	■	□	■	■
FDS 2		■			■	■	■					■	■	■		■
FDS 3			■	■	■			□	■	■	■	■	■	■	■	■
FDS 4			■	■				□	■	■	■	■		■	■	■
FDS 5								■						■	■	■
FDP 2												■	■	■		■
FDP 3						■								□	■	□
FDP 4								□							■	□
FDP 5																
aU										■	■					
aR																■
DASH	56	14	4	10	8	7	35	1	35	18	9	13	63	21	13	24
CISS	80	36	21	54	54	18	36	7	62	50	22	33	53	28	17	48
Inj side	R	R	L	R	L	L	R	L	L	L	L	L	L	L	L	R
Age at inj	12	33	34	36	38	13	35	57	60	36	34	17	53	9	34	36
Time from inj	8	16	15	9	6	6	11	5	4	3	11	17	8	9	5	2

Structures injured in addition to the median nerve injury, each of the participant represented in columns and sorted based on number of injured structures. ■ = completely transected structure, □ = partially transected structure, x denotes bilaterally missing tendon. Lower part of table shows scores from questionnaires regarding subjective symptoms after injury. Notably, there is no apparent correspondence between number of injured structures and the scores in the questionnaires. BR = Musculus brachioradialis, FCR = m. Flexor carpi radialis, PL = m. Palmaris longus, FCU = m. Flexor carpi ulnaris, FPL = m. Flexor pollicis longus, FDS = m. Flexor digitorum superficialis, FDP = m. Flexor digitorum profundus, 2–5 denotes digit specific tendons originating from these muscles, aU = Ulnar artery, aR = Radial artery. DASH = Disabilities of Arm, Shoulder and Hand score, CISS = Cold Intolerance Symptom Severity score (questionnaire 2). Inj side = side of forearm of median nerve injury. Age at inj = age in years at time of injury and surgical repair. Time from inj = the time in years from injury to the time of the study.

The nerve-injured participants appeared quite homogeneous regarding the clinical outcome of the injury. None of the injured participants had evident sudomotor dysfunction or constraints in range of motion of the affected hand. Nevertheless, all injured participants showed a modest thenar muscle atrophy of the affected hand and, in agreement with previous reports on corresponding injuries^38^, all had impaired static 2-point discrimination threshold (>7 mm) at the tips of the reinnervated digits.

To assess the practical impact of the injury, in the format of a nondirective interview we asked the participants to describe how the injury affected their everyday life. This interview was followed by structured questionnaires (see further below). From the interview, again a quite homogenous picture emerged. In agreement with previous reports on consequences of median nerve injury^4^, they all reported clumsiness and difficulties in tasks that require fine finger dexterity. Several activities were described as impossible to perform, such as buttoning a shirt without sight. Some of the participant reported accidentally burning, cutting or squeezing their injured hand because of impaired sensation. Furthermore, all participants indicated an increased dependence on vision especially when performing fine manual task, such as picking up small objects, tying shoelaces and handling cutlery. Symptoms connected with cold intolerance were also described, including freezing more quickly, color changes, stiffness, and reduced dexterity. Nevertheless, all of the nerve-injured participants were working at the time of the study and those within working age at the time of injury returned to their previous work after rehabilitation. Various professions were represented, such as truck driver, bus mechanics, telecommunications assembler, engineer, carpenter, warehouse worker, process operator, and nursing assistant.

For the questionnaires, the injured participants completed the Swedish versions of the ‘Cold Intolerance Symptom Severity’ (CISS) questionnaire and the ‘Disabilities of the Arm, Shoulder and Hand’ (DASH) questionnaire (for details see Methods). For these questionnaires higher scores indicates higher degree of impact of the injury. The CISS- and DASH-scores were similar to outcomes previously reported for persons with corresponding injuries and treatment regimens^39,40^ (CISS = 36/39 ± 20; DASH score = 13/21 ± 18 [median/mean ± 1 s.d.]; bottom panel in Table 1). For the right- and left-side injured groups the median CISS-score was 48 and 28, respectively. The corresponding data for the DASH score was 24 and 13. Although the right hand injured participants tended to report higher scores in both these questionnaires, these differences between right and left hand injured participants were no statistically significant (CISS, P = 0.13; DASH, P = 0.11; Mann-Whitney U test).

### Structural changes in the brain after median nerve injury

**Figure 1:**
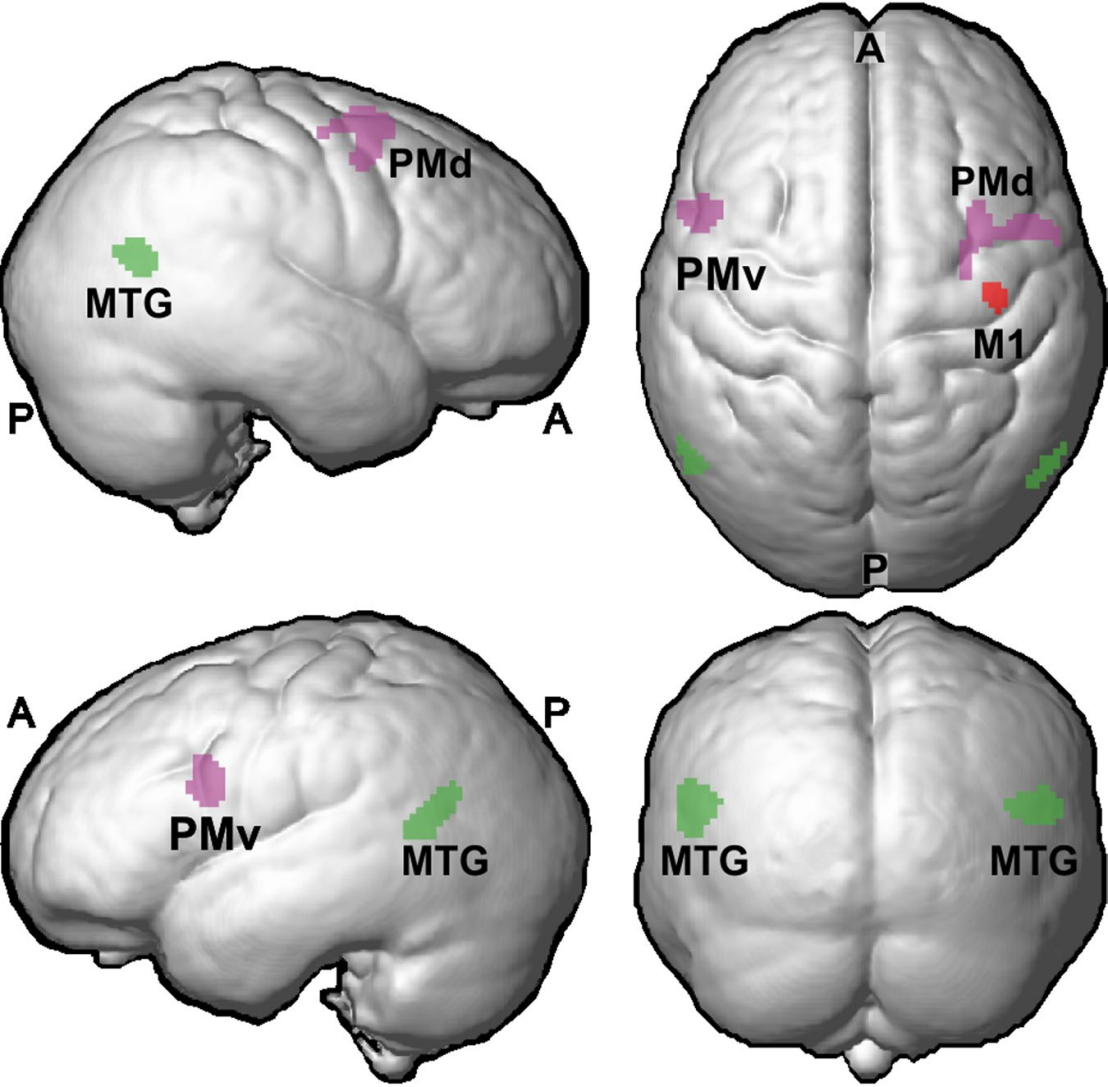
Overview of cortical areas showing effect of median nerve injury. Magenta indicate premotor areas with reduced gray matter volume as compared to control participants (PMd, dorsal premotor cortex; PMv, ventral premotor cortex) and green indicates extrastriate visual areas with increased gray matter volume (MTG, middle temporal gyrus). Red indicates reduced gray matter volume in primary motor cortex (M1). Data averaged across participants and rendered on an average brain based on all participants in the study (n = 32) created using SPM8’s ‘Create Rendering/Surface’-function with DARTEL template in MNI-space (render depth, 20 mm; L, left; R, right; A, anterior; P, posterior).

Figure 1 gives an overview of cortical areas where the median nerve injury significantly affected the gray matter volume (for statistical criteria see Methods). Such effects were present in premotor cortex, extrastriate visual cortex and primary motor cortex. In addition, we found significant effects in white matter pathways that connect the premotor cortices of the two hemispheres.

#### Gray matter effects in primary sensorimotor cortex

**Figure 2:**
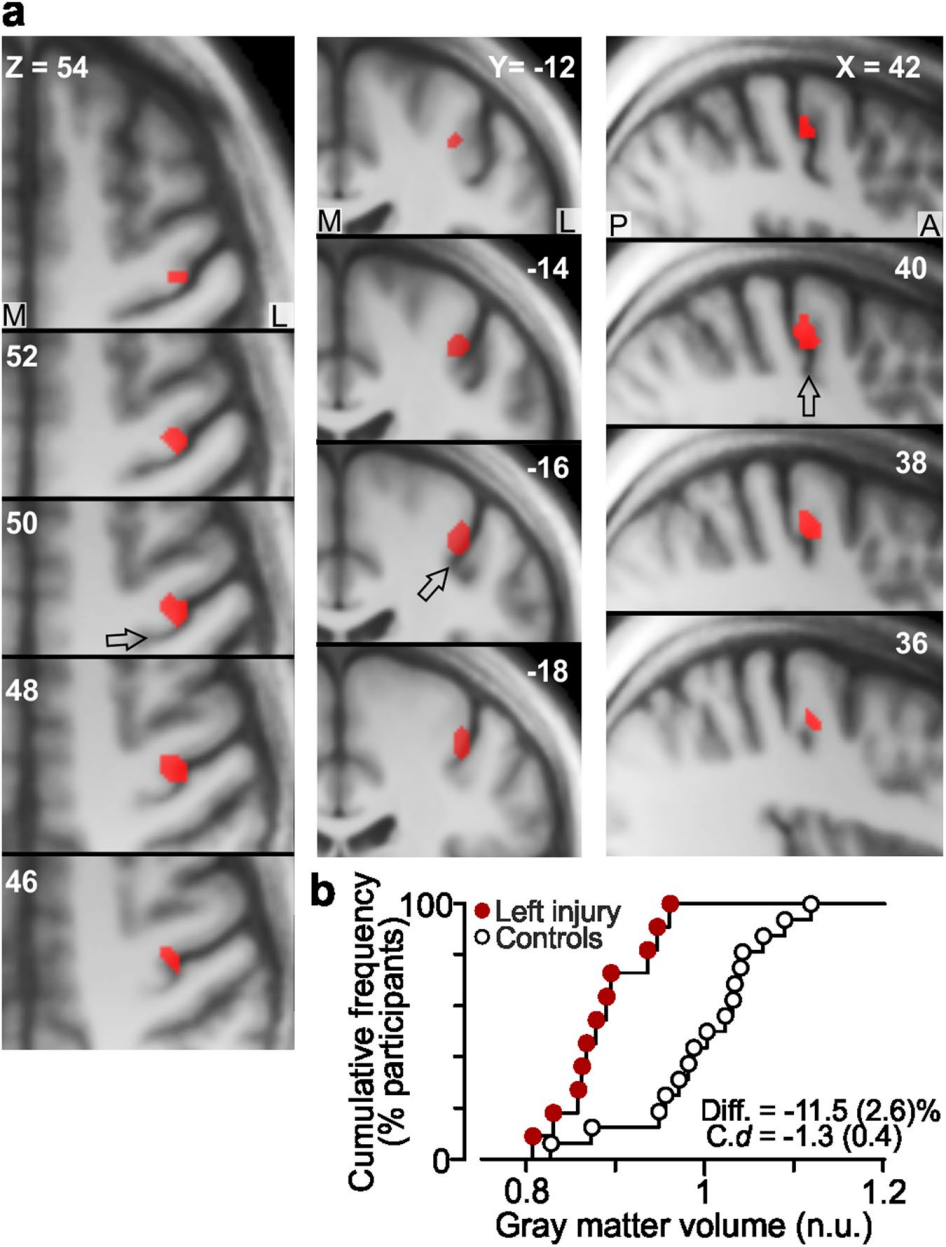
Reduced gray matter volume in the primary motor cortex contralateral to the affected hand. (**a**) Decreased gray matter volume (46 voxels) in left-sided injured participants compared to control participants in the right precentral gyrus (local maximum, X, Y, Z = 40, −16, 50; t_25_ = 3.4) revealed by SVC-analysis with left-sided injured patients. Effect (red) shown on axial, coronal and sagittal slices of the averaged brain calculated across the participant-specific T1-weighted images (n = 32) after being normalized to the MNI brain template. M, medial; L, lateral; A, anterior; P, posterior. Arrows denote central sulcus. (**b**) Distribution of gray matter volume for the left-sided nerve-injured participants (red filled circles) and for the control participants (black open circles) in the detected zone normalized to the mean value of the controls (n.u., normalized units; see Methods). “Diff”. indicates effect size given as mean difference (and standard error) in percent between the volumes obtained for the injured and the control group and “C. *d”* gives standardized effect size provided as Cohen’s *d* (and standard error of the effect size estimate).

For the SVC analysis of the contralesional primary sensorimotor cortex (S1 and M1), we used a sphere with a diameter of 20 mm centered on the MNI-coordinates of the centroid for the hand area of the primary sensorimotor cortex as established in a meta-analysis^41^. For the left-side injured participants, our SVC analysis detected reduced gray matter volume in a limited zone (46 voxels) in the anterior bank of the contralesional right central sulcus (BA 4; Fig. 2a). More specifically, the effect was located at the lateral convexity of the knob-like structure of the precentral gyrus, which is regarded as a reliable anatomical landmark of the hand area of the M1^42^. The gray matter volume was reduced by on average 11.5% compared to the control participants and the standardized effect size was -1.3 (Cohen’s *d*; s.e.m. = 0.4).

A corresponding analysis for the right-side injured participants failed to detect any effect in the contralesional primary sensorimotor cortex. However, a greater inter-individual variability in the location of the hand representation in the left than in the right hemisphere (possibly remaining after the spatial normalization procedure) could have prevented detection of an effect in the left primary sensorimotor cortex. To address this issue, we repeated the SVC-analysis after realigning the smoothed brain image of each participant in the transaxial plane based on the coordinates of the cortical knob-like structure. To that end, we used the non-smoothed normalized gray matter images and focused on the transaxial slice where we observed the strongest effect in the right M1 (Z = 50 mm; Fig. 2a). In this slice, we identified for each participant and hemisphere the apex of the omega shaped cortical knob-structure and used the recorded X and Y coordinates for the realignment. Defined as the Euclidian distance of the sampled coordinates to their centroid, the inter-individual variability compensated for by the realignment was 1.4 ± 0.2 and 1.6 ± 0.2 mm (mean ± 1 s.e.m.) for the left and the right hemisphere, respectively. We found no statistical difference in the inter-individual variability between hemispheres (left, right) and across group of participant (left injured, right injured, control): A mixed design ANOVA failed to show significant main effects of hemisphere (F_1,29_ = 0.14, P = 0.71) and group (F_2,29_ = 1.8, P = 0.19) and there were no significant interaction effects between these factors (F_2,29_ = 3.0, P = 0.07).

After the realignment, the SVC-analysis still failed to detect any significant effect for the right-side injured participants. Although, this analysis clearly suffered from low power since it was based on only five injured participants, no voxel was detected even with a very liberal threshold criterion involving a voxel-based threshold of Z >1.96 (P < 0.05) and no cluster extension threshold. Notably, the gray matter effect observed in the right M1 hemisphere of the left injured participants in the original SVC-analysis (46 voxels; local maxima t_25_ = 3.4) was enhanced after the realignment (83 voxels; local maxima t_25_ = 4.3), which suggested that our realignment procedure helped in controlling for inter-individual variation in the location of the hand representation. Corresponding SVC analyses of the ipsilesional primary sensorimotor cortex for each group of injured participants did not reveal any significant effect.

To investigate possible relationships between the functional impact of the injury in a subjective perspective and the size of observed structural changes, we correlated the DASH-scores with the standardized effect size for the area showing a structural effect in the right M1 of the left injured participants. We neither found a significant correlation for this area in the SVC analysis performed before nor after the realignment (both instances r^2^ < 0.06, P > 0.46).

#### Gray matter effects in premotor cortex and related white matter changes

The whole brain analysis that contrasted the left- and the right-side injured participants (n = 11 and 5) revealed no gray and white matter effects related to the side of injury. The failure of this analysis to reproduce the 46-voxel gray matter effect in the right M1 observed in the SVC-analyses could be explained by a higher cluster threshold value in the whole brain analysis (185 voxels).

**Figure 3:**
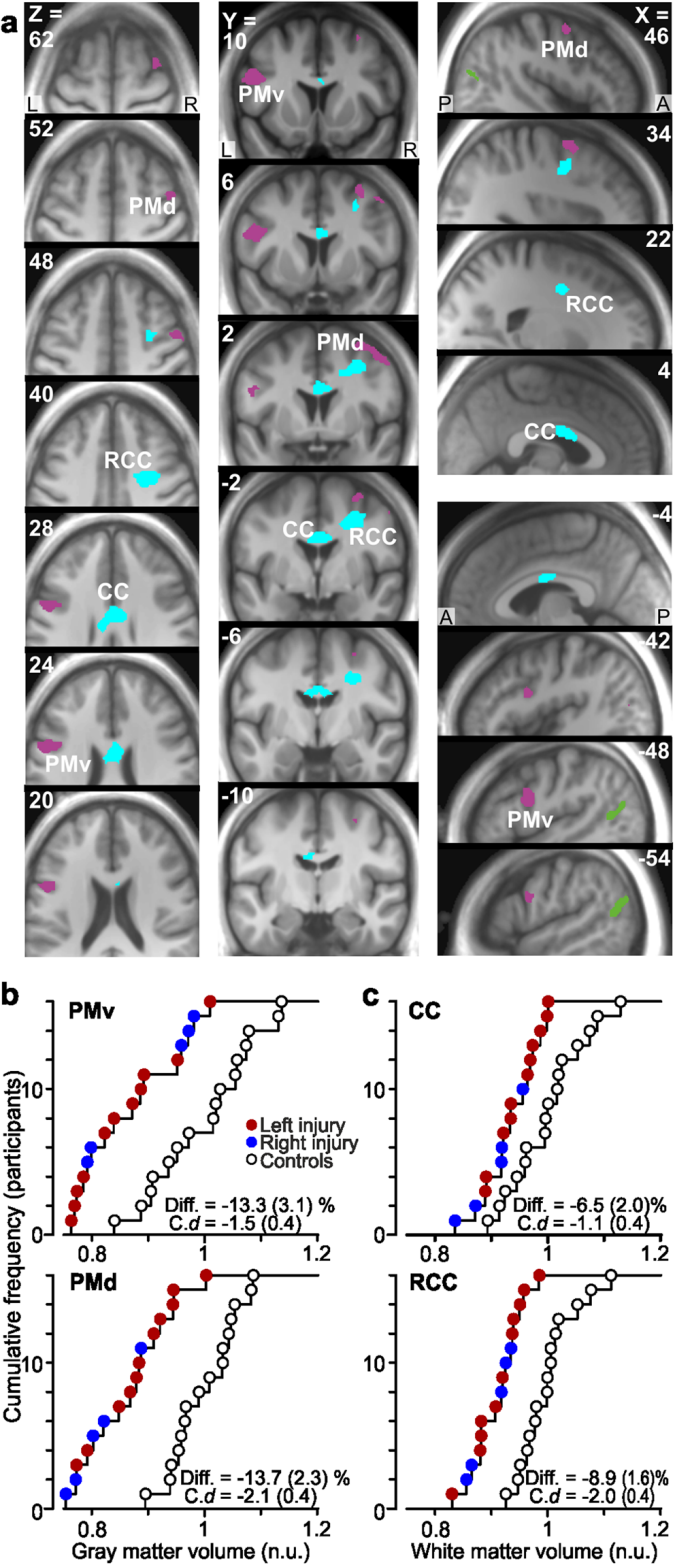
Reduced gray and white matter volume in premotor areas of median nerve-injured participants. (**a**) Areas with reduced gray (magenta) and white (cyan) matter volume as compared to control participants shown on axial, coronal and sagittal slices. (Green areas indicate extrastriate visual areas with increased gray matter volume). For the gray matter, one cluster (206 voxels) with a single local maximum (−48, 6, 26: Ze = 3.8) was located in the ventral premotor cortex (PMv) of the left hemisphere and one cluster (207 voxels) showing three local maxima (34, 2, 62: Ze = 3.6; 46, 2, 52: Ze = 3.0; 30, −12, 56: Ze = 2.7) was located in the dorsal premotor cortex (PMd) of the right hemisphere. For the white matter one cluster (276 voxels) with two local maxima (6, 0, 28: Ze = 3.1; −6, -8, 26: Ze = 3.0) was located in the mid-body of corpus callosum (CC) and one cluster (301 voxels) with a single maximum (32, 0, 40: Ze = 4.3) was located in the white matter beneath the right PMD radiating from corpus callosum (RCC). R, right; L, left; A, anterior; P, posterior. (**b**) and (**c**). Distribution of gray matter volume in the detected zones in left PMv and right PMd, and white matter volume in CC and RCC (from areas in **a**) among the nerve-injured participants (red and blue filled circles) and the control participants (black open circles). For further details, see legend of Fig. 2.

In the subsequent whole brain analysis, we contrasted all nerve-injured participants and the control participants (n = 16 and 16) to seek for effects common to the nerve-injured participants irrespective of injured side (left, right). As for the whole brain analysis that contrasted the left- and right-side injured participants, no effects were observed in the primary sensorimotor cortices, but effects were detected in other brain areas. First, we found reduced gray matter volume in two lateral premotor regions. This involved a zone of the left ventral premotor area (PMv) in the ventral sector of the left precentral sulcus, at the junction between Brodmann area [BA] 6 and Broca’s area (BA 44), and a zone of the right dorsal premotor area (PMd) in the precentral and frontal middle gyrus (BA 6; see Figs 1 and 3a, areas in magenta). For both areas, the injured participants showed a reduced gray matter volume by on average around 13% compared to the control participants and the Cohen’s *d* was −1.5 (s.e.m. = 0.4) and −2.1 (0.4) for PMv and PMd, respectively (Fig. 3b). Median nerve injury had no detectable effects on the gray matter volume in the homologous contralateral areas, i.e., the right PMv and the left PMd.

**Figure 4:**
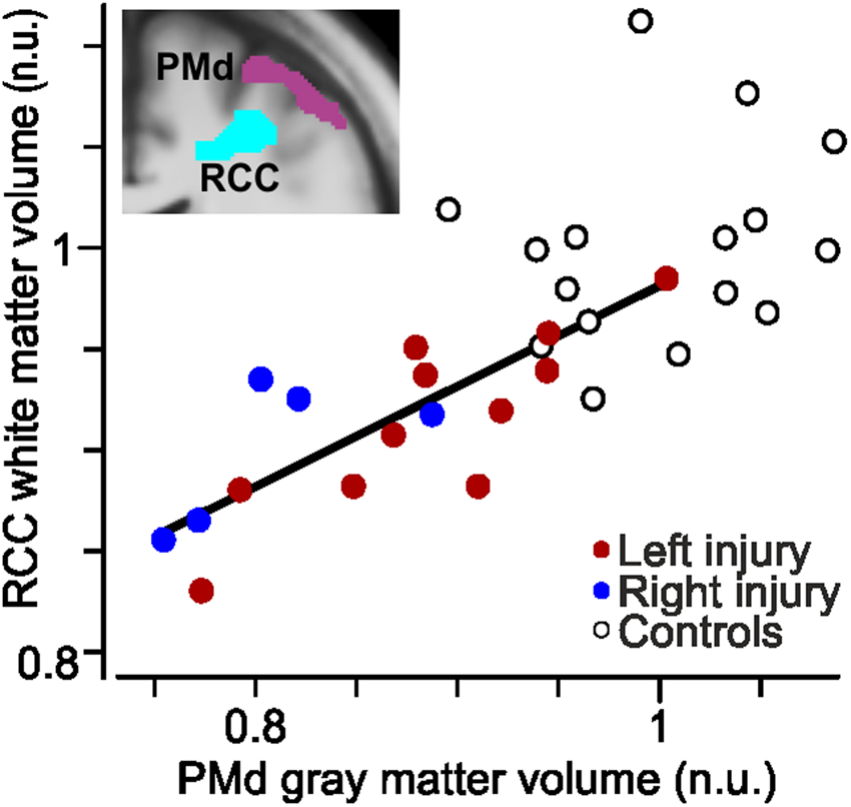
Correlation of gray and white matter volumes in right premotor areas. Positive correlation between the gray matter volume in the right dorsal premotor cortex (PMd) and the white matter volume in the right radiation of corpus callosum (RCC) across the nerve-injured participants. The solid line shows the orthogonal linear regression line; participants with left (red) and right (blue) sided injury pooled. No corresponding correlation was present for the control participants (black open circles, r = 0.23, t_14_ = 0.90, P = 0.37). Inlay of slice image from Fig. 3a. (Y = 2) to illustrate anatomical proximity of gray matter effect in the right PMd and white matter effect in RCC. n.u., normalized units (see Methods).

Second, the nerve-injured participants also showed volume reductions in white matter tracts (Fig. 3a, areas in cyan). Specifically, this reduction was observed in the midbody of the corpus callosum (CC) that interconnect the premotor areas of the two hemispheres^43,44^ and in a sector of white matter radiating from corpus callosum (RCC) to the right PMd. The Cohen’s *d* was −1.1 (s.e.m. = 0.4) and −2.0 (0.4) for these zones, respectively (Fig. 3c). Amongst the nerve-injured participants, the decrease in volume of the white matter radiation correlated with the decrease in gray matter volume in the right PMd (r = 0.77, t_14_ = 4.6, P < 0.001; Fig. 4).

The left- and right-side injured groups showed similar structural effects in the abovementioned premotor areas and white matter tracts (see red and blue filled circles in Fig. 3a and b). For the left PMv the standardized effect size was −1.7 (s.e.m. = 0.5) and −1.1 (0.5) for the left- and the right–injured groups, respectively. The corresponding values for the right PMd were −1.8 (0.5) and −3.5 (0.7), for the CC −0.9 (0.4) and −1.6 (0.6), and for the RCC −1.8 (0.5) and −2.2 (0.6). Although the standardized effect size appeared greater for right-side injured participants especially for PMd and CC, comparisons of effect sizes in these four areas between the left- and right-side injured groups failed to indicate any statically significant difference after Bonferroni correction for multiple comparisons (in all instances t_14_ <2.0, P_corrected_ >0.13). We found no significant correlation between the effect size in any of these four areas showing structural effect and the impact of the injury represented by the DASH-score (in all instances r^2^ <0.12, P > 0.18).

#### Gray matter effects in extrastriate visual cortex

**Figure 5:**
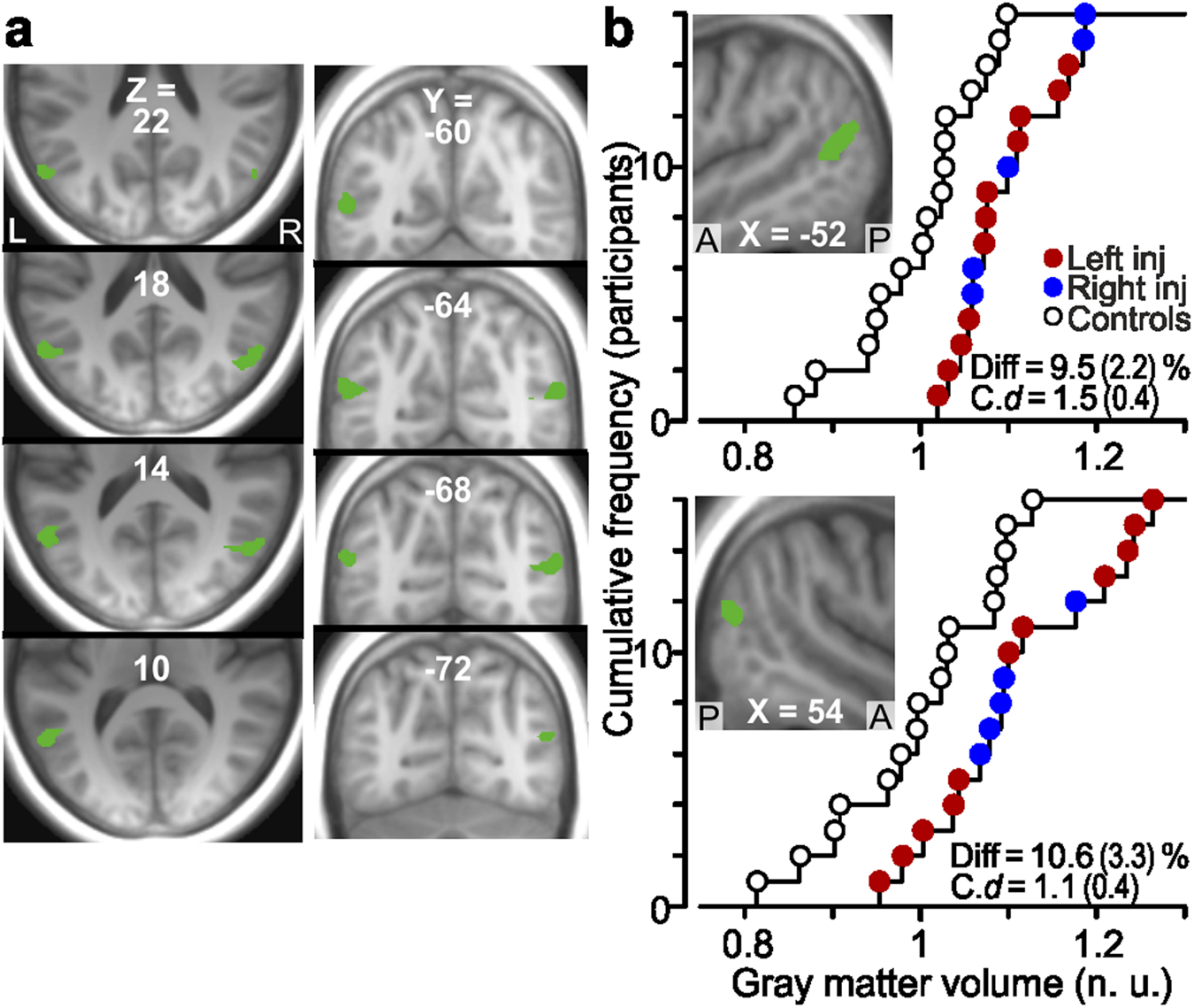
Increased gray matter volume in extrastriate visual areas of median nerve-injured participants. (**a**) and (**b**) Areas (green) with increased gray matter volume posteriorly in the left (180 voxels; local maximum −52, −64, 16: Ze = 4.4) and right (139 voxels; 54, −66, 16; Ze = 3.7) middle temporal gyrus of the injured participants compared to the control participants shown on axial, coronal and sagittal brain slices obtained as in Fig. 3a. Note the bilateral symmetry of the effect with a distance of only ~3 mm between the local maxima after mirror reversing one of the clusters across the mid-sagittal plane. (**b**) Distribution of gray matter volume in the detected zones in left (top graph) and right middle temporal gyrus (bottom graph) for the nerve-injured participants and the control participants. For further details, see legend of Fig. 3.

In the nerve-injured participants we also observed regional increases in gray matter volume, namely bilaterally in the posterior part of middle temporal gyrus near the occipito-temporal junction close to the intersection of BA 39, 37 and 19 (Fig. 5a). We attribute the localization of this effect to the medial superior temporal area (MST) in the anterior-superior zone of the human homolog of the macaque motion complex (V5/hMT+) of the visual cortex^45,46^. For the injured participants regarded as one group, the Cohen’s *d* for the left and right hemisphere was 1.5 (s.e.m. = 0.4) and 1.1 (0.4), respectively (Fig. 5b). Similar effect sizes were found when analyzing the left- and right-side injured participants separately (see red and blue symbols in Fig. 5). For the left hemisphere, the effect size was 1.4 (s.e.m. = 0.4) for left injured and 1.7 (0.6) for right injured. The corresponding values for the right hemisphere were 1.1 (0.4) and 1.2 (0.5). Comparisons of standardized effect sizes between the left- and right-side injured groups in these two areas failed to reveal any statically significant difference (in both instances t_14_ < 0.7, P > 0.25). We found no significant correlation between the DASH-scores with the effect size in any of these areas (r^2^ < 0.21, P > 0.07).

## Discussion

Our findings indicate that median nerve transection followed by surgical repair and reinnervation of the hand can cause long-term structural changes in several brain areas, and in particular those that are functionally implicated in planning and control of skilled manual actions. That the detected effects were synaptically remote from the lesioned neurons and involved reductions as well as increases of cortical gray matter volume strongly suggest that they represent complex activity-dependent adaptations in specific processing units in the brain^11,12,47,48^.

Strikingly, the observed structural changes in the brain predominantly concerned higher-order rather than primary sensorimotor areas. The lack of detectable effect in contralesional S1 and limited effects in contralesional M1 stands in contrast to the considerable gray matter effects in these areas reported in persons with sensorimotor deprivation caused by amputation or immobilization of an upper extremity^18–20^. A likely explanation for the overall spared primary sensorimotor areas in our nerve injured participants is that they regularly used their affected hand in everyday activities. Indeed, the consequences of the injury were subtle enough for them to return to their usual jobs. Nevertheless, our nerve injured participants were unable to perform certain everyday tasks requiring fine finger dexterity, such as tying shoelaces. This behavioral restriction can be explained by the nerve injury preventing them from successfully executing one or more of the sequentially linked action phases involved in the performance of the impeded tasks^49^. We believe that the decrease in the gray matter volume detected in higher-order sensorimotor areas (PMv, PMd) reflected decreased neural processing associated with such limitations in the normal manual action repertoire. For the effect found in extrastriatal visual cortex of the nerve-injured participants, we believe that the gray matter increase reflected increased visuomotor processing for compensation of a compromised tactile sensory innervation of the affected hand.

For the primary sensorimotor cortex, we found gray matter volume reduction in a limited zone of the contralesional M1 for the left-side injured participants but not for the right-side injured. This suggest that the activity in the left M1 after contralateral injury is maintained to a higher degree than the corresponding activity in the right M1. At least three different factors related to hemispheric specialization in the human brain of right-handers might contribute to such an asymmetry in M1 activity. First, compared with the left-side injured, participants with their right dominant side injured were probably more inclined to reclaim the use of the affected hand after reinnervation and would therefore have engaged the contralesional M1 to a higher degree. The second factor relates to the observation that the engagement of the ipsilateral M1 differ for left and right hand actions. That is, left hand actions engage the left ipsilateral M1 to a higher degree than right hand actions engage the right ipsilateral M1^50,51^. Therefore, left hand actions performed by the right-side injured would tend to maintain processing in the left M1 to a higher degree after the injury than right hand actions performed by left-side injured would tend to do in the right M1. Third, a restriction in the bimanual action repertoire due to the nerve injury is likely to reduce the processing in the contralesional M1 more in the left- than in the right-side injured participants. This is because the use of the left-hand, in relative terms, is more often associated with bimanual tasks compared to the use of the right dominant hand. Indeed, complex bimanual coordination involving fine finger dexterity characterizes most daily manual skills, such as dressing, personal hygiene, grooming, preparation of food and eating, and numerous work-related tasks often involving tool usage^52,53^.

The lack of activity-dependent gray matter effects in the contralesional S1 irrespective of injured side is not entirely surprising since functional imaging studies in humans have reported that tactile stimulation of fingers can activate the contralateral S1 to a similar extent after median nerve injury and reinnervation as in healthy individuals^17,32,33^. Likewise, experimental studies in monkeys have shown that the median nerve largely recaptures its original cortical territory after transection and reinnervation, but with distortions of the somatotopic representation^24–26,48^.

We suggest that the reduced gray matter volume detected in PMv and PMd of the nerve-injured participants were associated with decreased neural processing in these areas due to the restrictions in their natural manual action repertoire (see above). Through mutual interactions and interactions with M1, these premotor areas are responsible for higher-level planning and control of goal directed manual actions. To that end, they integrate information about action-goals from prefrontal cortex and multimodal sensory state information from parietal cortex, including somatosensory information from the hands^54–60^. That the effects observed in the premotor areas were lateralized in a hemispherical asymmetrical manner independent of side of injury is in accordance with evidence for hemispherical specialization in higher-level multi-modal processing that underlie implementation of learned skilled hand actions in right-handers^34–37^.

The left PMv, located at the junction between premotor area 6 and Broca’s area 44, is considered to play a crucial role in the transformation of mental representations of complex manual action goals into action elements of different degrees of abstraction in right-handers^54,61–63^. Accordingly, the reduction in the gray matter of the left PMv irrespective of side of injury likely reflected a reduced, or even ceased, processing in certain neuronal assemblies representing compound motor acts that could not be realized because of the insufficient innervation of the affected hand. In the same vein, a reduction of the gray matter lateralized to the right PMd irrespective of affected hand is consistent with a sub-population of PMd-neurons showing limb-independent processing^64,65^ and that the right PMd has a special role in the kinematic processing of grip formations in humans irrespective of acting hand^66^. Similarly, dorsal parieto–premotor networks of the human right hemisphere are considered implicated in the processing of kinematic aspects of hand movements in haptic object exploration^67^ and, more generally, in processing of spatial aspects of occurrences across and close to the entire body surface^68,69^. Moreover, since the right PMd seems particularly involved in complex bimanual coordination^70–72^, restrictions in the bimanual action repertoire due to impairment of one hand could have contributed to an activity-dependent reduction of gray matter. In accordance with findings on activity-dependent changes in myelination^12^, the observed white matter reduction in the premotor commissural pathways would also be in agreement with restrictions in the bimanual action repertoire.

The gray matter increase observed bilaterally in the posterior middle temporal gyrus of the nerve-injured participants matched the increased dependence on vision in activities requiring skillful object handling at impaired tactile innervation of a hand^4,73–75^. The localization of the effect to the V5/hMT+ complex agrees with previous observations that this area constitutes a node in the parieto–premotor pathway of the dorsal visual stream that supports visually guided action^76–81^. Likewise, manual visuomotor skill acquisition is associated with increased gray matter density especially in this extrastriate visual area^13,82^. That the V5/hMT+ complex is considered as the earliest site in the parieto–premotor pathway where visual motion signals and tactile information are integrated^46,83–86^ suggest that the increase in gray matter in this area reflected an intensified cross-modal processing for functional compensation of the somatosensory deficiencies of the affected hand.

To our knowledge, only one previous study has examined structural brain changes after injury and repair of the principal nerves innervating the hand^17^. This study applied voxel-based cortical thickness (VBCT) analysis and included 14 patients with right median and/or ulnar nerve injury. In contrast to our findings, this study only reported reductions in gray matter and in areas not specifically associated with hand control, including areas of post-central gyrus seemingly distant from the primary hand representations. Methodological factors presumably account for this difference: VBM and VBCT measures different structural characteristics and are considered complementary for local gray matter analysis, where VBM is additionally sensitive to local surface area and cortical folding^87–89^. In addition, the participant-groups in the two studies were heterogeneous regarding nerves injured.

Our results are limited by several factors, including those inherent to the VBM technique^90^. Although the restricted sample size limited the statistical power of relevant analyses, the effects exposed in the premotor and the extrastriatal areas were seen in both the left- and right-side injured subgroups when analyzed separately. That the detected structural effects only included fractions of the network of brain areas and pathways implicated in control of manual dexterity suggests that the identified areas were the most sensitive ones concerning activity-dependent structural plasticity associated with persistent dexterity impairments of the nerve-injured participants. Another weakness in our study concerns deficiencies in the characterization of the behavioral impairments of our nerve-injured participants, which could have prevented the detection of possible relationships between sizes of structural effects and functional impacts. Neither established questionnaires, such as the DASH questionnaire, nor existing performance tests, which focus on what a person can do in a highly standardized environment, address systematically the natural diversity and complexity of fine finger dexterity at a level of detail required for quantification of the various more or less subtle aspects of the disabilities remaining after median nerve reinnervation^91,92^. Likewise, established performance tests do not explicitly address bimanual behaviors. Moreover, the kind of cross-sectional study used here does not favor the discovery of quantitative relationships between behavioral and structural effect sizes. First, since the structural conditions of the individual brain prior to injury were not available as a reference, the amount of structural change after injury could not be assessed at the individual level. Second, our study reveals little about the time course of structural brain changes after median nerve injury. Additional gray and white matter changes may have existed during the months-long period when the hand was partially paralyzed and deprived of sensations before reinnervation^7,22^. Depending on the degree of reengagement of the hand in daily assignments after reinnervation, some structural changes might have reversed totally whereas others only partially^47,93,94^. Resolution of both of these potentially confounding factors would require a longitudinal study design that maps the structure of each brain before it is affected by the nerve injury.

## Methods

### Participants

The study included 16 median nerve-injured right-handed adult participants (four females) and 16 healthy right-handers matched to age and sex. None of the participants suffered from diabetes or neurological disorders. All participants gave their informed written consent in accordance with the Declaration of Helsinki. The ethical committee of Umeå University approved the study. Eleven of the participants had suffered sharp complete transection injury of the median nerve at the left distal forearm (three females) and five (one female) at the right distal forearm. The distance between the tip of the index finger and the site of injury varied between 17 and 27 cm (mean = 20 cm) across the nerve-injured participants. All were treated at the University Hospital of Umeå and underwent microsurgical primary repair with epineural end-to-end suture of the injured nerve within 24 hours following the injury. Injuries to tendons and arteries (Table 1) were surgically repaired in the same session.

A standard clinical assessment of the nerve-injured patients was performed more than two years after surgery. This concerned sudomotor function, range of motion and muscle atrophy of the affected hand, and static 2-point discrimination threshold at the tips of the reinnervated digits. To assess the practical consequences of the nerve-injury, participants were asked to describe how the injury affected their everyday life including effects on working ability. All participants also completed the Swedish versions of the ‘Cold Intolerance Symptom Severity’ (CISS) questionnaire^4,39,95^, which indicates the severity of subjective post-injury symptoms elicited by exposure to cold, including pain, aching, numbness, stiffness, weakness, swelling, and change in skin color. It yields a score from 4 to 100 where >30 are considered abnormal^96^.

They also completed the ‘Disabilities of the Arm, Shoulder and Hand’ (DASH) questionnaire^97,98^, which evaluates the disability of the upper extremity on a scale from 0 to 100, where >10 is considered pathological^99^. The DASH questionnaire contains 30 items that scores the respondent’s self-experienced ability to perform certain upper extremities that are common in daily life. The questionnaire’s instructions to responders tells that “It doesn’t matter which hand or arm you use to perform the activity; please answer based on your ability regardless of how you perform the task”. Thus, the responses to the DASH questionnaire do not indicate how the activities are conducted and to what extent behavioral compensatory strategies are used to deal with the effects of the injury. For the healthy controls, the outcomes of these questionnaires was normal. Only two participants reported some cold intolerance where one scored 4 and the other 8 points. The DASH score was 0/0.7 ± 1.7 (median/mean ±1 s.d.).

### MRI parameters and image pre-processing

We acquired structural MRI-scans of the whole brain with a Discovery MR750 3 T scanner equipped with a 32-channel head coil (GE Medical Systems, Milwaukee, Wisconsin, USA). Sponges in the head-coil stabilized the participant’s head. We used a three-dimensional fast spoiled gradient echo sequence to acquire a series of 180 transaxial T1-weighted images with the following MR-parameters: TE, 3.2 ms; TR, 8.2 ms; flip angle, 12°. The field of view was 25 × 25 cm and contained a 512 × 512-pixel matrix, giving voxels of 1.0 × 0.49 × 0.49 mm. We manually aligned the collected MRI-scans of each participant using the ‘Display’ function in SPM8 (http://www.fil.ion.ucl.ac.uk/spm/), and then segmented the scans into gray matter, white matter, cerebrospinal fluid, skull, and air. To improve the accuracy of inter-subject alignment we used diffeomorphic image registration (DARTEL)^100^; of the segmented gray and white matter images before we normalized the single-participant images to MNI space. The option ‘Preserve Amount’ was chosen for the normalization, as suggested for VBM-analyses in SPM8. The images where then smoothed with an 8-mm full-width at half-maximum Gaussian kernel (final voxel size: 2 × 2 × 2 mm).

### Statistical parameter mapping (SPM) analyses

We then entered the pre-processed single-participant gray and white matter images into group analyses using the voxel-based morphometry (VBM) software provided in SPM8^21^. A general linear model analysis was performed at each voxel where group of participants constituted the predictor of prime interest. We used total intracranial gray and white matter volume as global nuisance effect both in the gray and white matter analysis (global normalization, ANCOVA in SPM8). Furthermore, to statistically control for influences of age and sex on regional gray and white matter volumes^101^, in all analyses, age constituted a continuous predictor and sex a categorical predictor. To protect against false positives while at the same time retain the power to detect meaningful effects in the face of multiple comparisons across image voxels^102^, in all statistical analyses of effects of nerve injury (see below) we subjected the preprocessed morphometric images to a two-step threshold approach (Forman *et al*., 1995). We combined a voxel-based threshold of P < 0.005 (Z-equivalent [Ze] >2.6) with a cluster size threshold of P < 0.05 corrected for family-wise error (FWE) rate. The cluster size threshold was determined based on random field theory^103–105^ and implemented with the stat_threshold function of fmristat (www.math.mcgill.ca/keith/fmristat/). In our statistical analyses, we chose not to increase artificially their power by pooling data from the left- and right-sided injured participants by reversing the x-coordinates for one of the groups (“flipping the brain”). Given that hemispheric functional asymmetries in the control of dexterity are evident both for brain areas linked to higher level planning and control of manual praxis^34–37^ as well as for primary sensorimotor cortex^50,51^, we reasoned that “flipping the brain” would prevent discovering possible hemispheric asymmetries regarding structural changes in the nerve-injured participants.

#### Small volume correction SPM analyses

To test specifically our hypothesis of a change in gray matter volume within the contralesional M1 and S1, we compared each group of injured participants (left- and right- side injury separately) with the control participants using a small-volume correction (SVC) analysis implemented in SPM8 (two-tailed t-test). The target volumes were two spheres, each with a diameter of 20 mm centered on the MNI-coordinates X = ±39, Y = −21, and Z = 54. We based these coordinates on average local maxima of the 3-D peak coordinates for the primary sensorimotor cortex of the hand established in a meta-analysis based on functional brain imaging^41^. To maximize the power we included all 16 control-participants.

#### Whole brain analyses

We also applied a whole brain analysis to seek for effects beyond the primary sensorimotor cortex. In these analyses, we looked for effects on gray and white matter that would depend on side of injury as well as effects that would be independent of side of injury. In a whole brain analysis, we contrasted the left- and right-side injured participants (n = 11 and 5, respectively) to search for effects depending on side of injury. We then searched for effects common to the injured participants, independent of affected hand, by comparing the injured participants as a group (n = 16) against the control participants (n = 16; F-test). For this analysis, a cluster size threshold of one-half of that in the standard double-threshold approach was used to search for areas showing symmetrically localized bilateral effects on the gray and white matter volume. We motivate this approach with the argument that bilateral homologous effects with high probability indicate meaningful effects given the strong structural and functional connectivity between bilaterally connected areas^43,44,106,107^.

### Descriptive statistics

To explore the signs and sizes of effects detected in the SPM analyses, we first extracted gray/white matter volumes within the identified areas. For each participant and area, we then normalized the measured gray/white matter volume to the global gray/white matter volume estimated for that participant by dividing the regional volume by the global volume (reported as “normalized units” in figures). We report the standardized effect sizes using Cohen’s *d*, which describes the effect size as the difference between the means of the compared groups divided by the pooled standard deviation. Effect sizes are also reported as the signed difference between the mean values obtained for the injured and the control group expressed as percentage of the mean value of the control group; the estimate of the standard error (SE) was based on the pooled variance of the two populations. In data analysis, we used an alpha level of 0.05. Bonferroni corrections were used to compensate for multiple comparisons.

The datasets analyzed during the current study are available from the corresponding author on reasonable request. However, in accordance with patient confidentiality, personal data from participants in the study will not be disclosed.
